# Adeno-Associated Viral Vector Serotype 5 Poorly Transduces Liver in Rat Models

**DOI:** 10.1371/journal.pone.0082597

**Published:** 2013-12-27

**Authors:** Paula S. Montenegro-Miranda, Astrid Pañeda, Lysbeth ten Bloemendaal, Suzanne Duijst, Dirk R. de Waart, Gloria Gonzalez Aseguinolaza, Piter J. Bosma

**Affiliations:** 1 Tytgat Institute for Liver and Intestinal Research, Academic Medical Center, Amsterdam, The Netherlands; 2 Centro de Investigación Medica Aplicada (CIMA), Pamplona, Navarra, Spain; University of North Carolina at Chapel Hill, United States of America

## Abstract

Preclinical studies in mice and non-human primates showed that AAV serotype 5 provides efficient liver transduction and as such seems a promising vector for liver directed gene therapy. An advantage of AAV5 compared to serotype 8 already shown to provide efficient correction in a phase 1 trial in patients suffering from hemophilia B, is its lower seroprevalence in the general population. Our goal is liver directed gene therapy for Crigler-Najjar syndrome type I, inherited severe unconjugated hyperbilirubinemia caused by UGT1A1 deficiency. In a relevant animal model, the Gunn rat, we compared the efficacy of AAV 5 and 8 to that of AAV1 previously shown to be effective. Ferrying a construct driving hepatocyte specific expression of UGT1A1, both AAV8 and AAV1 provided an efficient correction of hyperbilirubinemia. In contrast to these two and to other animal models AAV5 failed to provide any correction. To clarify whether this unexpected finding was due to the rat model used or due to a problem with AAV5, the efficacy of this serotype was compared in a mouse and two additional rat strains. Administration of an AAV5 vector expressing luciferase under the control of a liver specific promoter confirmed that this serotype poorly performed in rat liver, rendering it not suitable for proof of concept studies in this species.

## Introduction

Efficacy in patients demonstrates that adeno-associated viral (AAV) vectors can be used for *in vivo* correction of inherited disorders. The large variety of AAV serotypes with different tropisms provides a gene therapy platform that can be applied in different tissues. For instance AAV serotype 1 (AAV1) can be used to correct lipase deficiency in the muscle [1], AAV2 and 8 both can restore factor IX expression in the liver [2], [3] and AAV2 effectively corrects inherited blindness [4].

Presence of low levels of neutralizing antibodies (NAb) towards an AAV serotype will preclude efficient transduction of the target organ *in vivo* [2], [5]. Therefore, the prevalence of these towards the different serotypes is highly relevant for their applicability as a gene therapy vector. A worldwide epidemiologic study shows that in the general population the seroprevalence for serotype 1 and 2 is the highest while for serotype 8 it is much lower [6]. Another study in healthy French donors revealed that the prevalence of NAb towards AAV8, 9 and especially AAV5 is the lowest [7]. The immune responses towards more than one serotype in an individual may in part be due to cross reaction because of the extensive homology between AAV capsid proteins. This seems a relevant mechanism since the amino-acid composition of the capsid of most serotypes differs only for 15% or less from that of AAV1 and 2, the two most prevalent serotypes. AAV5 is an exception since its capsid amino-acid composition differs by 40% [8]. The low co-prevalence of NAb towards AAV5 with that towards both AAV1 and 2 underscores the relevance of cross reactivity in the observed seroprevalence [7].

Our long term goal is to treat Crigler-Najjar syndrome (CN), familial severe unconjugated hyperbilirubinemia. This recessive inherited severe liver disorder is caused by deficiency of uridine diphospho-glucuronosyl transferase 1A1 (UGT1A1) [9], [10], a hepatic enzyme that catalyses the glucuronidation of bilirubin, an essential step in the excretion of this neurotoxic compound. Previously we have shown that AAV mediated liver-directed gene therapy for this disorder seems feasible. In the Gunn rat, a relevant animal model for CN syndrome, especially AAV1 provides efficient correction of serum bilirubin levels [11]. The high seroprevalence may however render this serotype not suitable for systemic administration in the majority of the CN patients. In this respect AAV8, 9 and especially AAV5 seem more promising. This and the very effective liver transduction by AAV 8 and 5 in non-human primates, indicates that of these two serotypes seem preferable candidates for *in vivo* gene therapy in CN patients. In addition, the availability of several suitable vectors does allow switching to another serotype for treating patients with pre-existing NAb towards one of these serotypes [12]. Furthermore, due to the episomal persistence of AAV vectors the correction may diminish in time. For a second treatment a different serotype is needed since due to the generation of NAb the efficacy of the initial serotype will be lost [13]. Since in mice, re-administration of another serotype does indeed allow efficient transduction at least of brain and muscle cells this seems a valid approach [13], [14].

In this study we evaluate the efficacy of scAAV5 and scAAV8 to that of scAAV1 in the Gunn rat. AAV8 and AAV1 both provide efficient correction of hyperbilirubinemia. In contrast, AAV5 does not provide any detectable activity in this rat model. Subsequent studies in different mouse and rat models using other transgene cassettes confirm the lack of efficacy of AAV5 in rat liver rendering proof of concept studies in this species with this clinically relevant serotype impossible.

## Materials and Methods

### Construction and production of AAV vectors

AAV vectors were constructed by replacing the factor IX cDNA with the UGT1A1 cDNA, using the EcoR1 and Bbs1 sites of plasmid scAAV-LP1-hFIXco (kindly provided by Dr. A. Nathwani, University College London, London, United Kingdom). This plasmid carries the AAV2 backbone with an intact 5′ terminal resolution site (trs) without the 3′trs analogous, a LP1 promoter consisting of core liver-specific elements from human apolipoprotein hepatic control region (HCR) and the human alpha-1-antitrypsin (hAAT) promoter, a modified SV40 small intron and the SV40 late polyA sequence, as previously described [15]. The plasmid was sequenced to confirm proper insertion of UGT1A1. AAV expressing luciferase under the control of the liver specific promoter composed by the albumin enhancer and the α1-antitrypinsin promoter (AAV-AlbEnhAAT-luc) was constructed as described [16].

Recombinant AAV was produced with AAV2 Rep and pseudotyped with capsid from AAV serotype 1, 5 and 8 using the adenovirus-free method described before [11]. AAV vector particles were purified by iodixanol gradient centrifugation as described [17]. Titration of AAV vectors was performed by quantitative PCR as described [11]. For this study three to five different virus preparations were made per AAV vector. The average yields of both AAV vectors varied between 3×10^11^ and 9×10^11^ genome copies per plate. Of the average titers (genome copies/ml) of the purified batches of the vectors were 5.3×10^12^ for scAAV2/1, 5.3×10^12^ for scAAV2/5 and 1.9×10^12^ for scAAV2/8. Because of the double stranded genome, each scAAV vector was calculated as containing 2 copies of ss viral genomes.

### Animal experiments

Gunn rats from our own breeding colony were used for all experiments and fed ad libitum. All animal experiments were performed in strict accordance with the Animal Ethical Committee guidelines of the Academic Medical Center of Amsterdam and the Animal Ethical Committee guidelines of the University of Navarra. The protocol was approved by the Animal Ethical Committee of the Academic Medical Center of Amsterdam and the Animal Ethical Committee of the University of Navarra.

Male Gunn rats, 8 to 10 weeks of age, with a weight between 180 and 200 g, received a single intraportal injection of 3×10^11^ gc/kg of scAAV-LP1-UGT1A1 vector packaged with each of the viral capsids from AAV1, 5 and 8. For portal injections the rats were anesthetized with an intraperitoneal injection of KAR mix: 4 ml ketamine (100 mg/ml), 2 ml Rompun (xylazine; 20 mg/ml), 1 ml atropine (1 mg/ml); dose of 0.1 ml/100 g body weight. Under deep anesthesia, the peritoneal cavity was opened and AAV vector resuspended in a maximum volume of 500 µl of PBS was injected into the portal vein using a 30-gauge needle. The animals were sutured and received the analgesic Temgesic subcutaneously following recovery from KAR mix. For bile collection, rats were anesthetized by intraperitoneal injection of KAR mix as above and bile was collected by cannulation of the bile duct as described [11]. Animals receiving the AAV5 vector expressing luciferase were tail vein injected. The Bioluminescence imaging and Ex vivo luciferase activity analysis of mice and rats was performed as described in [18].

Luciferase activity is represented as photons/sec and RLU per miligram of total protein respectively.

Blood was collected by tail vein puncture under gas anesthesia in pediatric heparin tubes. After spinning down the cells the plasma was stored at −80°C until bilirubin measurement performed at the routine clinical chemistry department.

### Analysis of genomic DNA and mRNA

High-molecular-weight DNA from tissues was isolated by a sodium dodecyl sulfate-proteinase treatment and subsequently complexed with silica particles in the presence of guanidinium thiocyanate, as described in [19]. The isolation of total liver RNA was performed using Trizol (Invitrogen, The Netherlands). cDNA was made using Superscript3 (Roche) and 1 µg of total liver RNA and OligodT and a specific primer for 18S. Real time quantitative PCR (qPCR) to determine the mRNA level in liver and vector genome copy number in the DNA isolated from various tissues obtained at the time of necropsy and blood samples was performed in 96-well plates in the Roche Light Cycler 480 using SYBR green to detect the amplification products. In all PCRs 100 ng of genomic DNA was used. The primers used were specific for human UGT1A1 and did not amplify rat UGT1A1: forward, 5′-GACGCCTCGTTGTACATCAG-3′; reverse, 5′-CACGCTGCAGGAAAGAATC-3′, using the following conditions 95°C for 5 min and 45 cycles of 95°C for 10 s, 60°C for 10 s, and 72°C for 12 s. The values were normalized with the rat β-actin gene, using as a forward primer: 5′ AGCCATGTACGTAGCCATCCA3′ and 5′ TCTCCGGAGTCCATCACAATG3′ as the reverse primer. A standard curve was generated by dilution in rat genomic DNA of the UGT1A1 plasmid. To determined the UGT1A1 mRNA levels in liver the qPCR was performed using the same primers for UGT1A1 as used to determine genomic copies and primers for 18S (Fw: 5′-CGAACCTCCGACTTTCGTTCT-3′ and a Rv: 5′-TTCGGAACTGAGGCCATGAT-3′). Vector genomic copies per diploid genome equivalent (vc/dGE) and UGT1A1 mRNA/18S were calculated using the LinRegPCR software [20].

### Bilirubin quantification

Total bilirubin in serum was determined by the routine clinical chemistry department using a standard colorimetric assay. Unconjugated bilirubin and bilirubin conjugates in bile were analyzed and quantified by HPLC as described [21] with the modification that an Omnisphere column (Varian, The Netherlands) was used [11].

### Statistics

The results obtained in C57/BL6 mice are statistically significant compared to the obtained in Sprague Dawley and Wistar rats using the nonparametric Mann–Whitney test (p<0.05 *, p<0.01 **, p<0.001 ***).

## Results

### In Gunn Rats scAAV Serotype 8 is more Efficient than 1 While scAAV 5 Completely Lacks Efficacy

**Figure 1:**
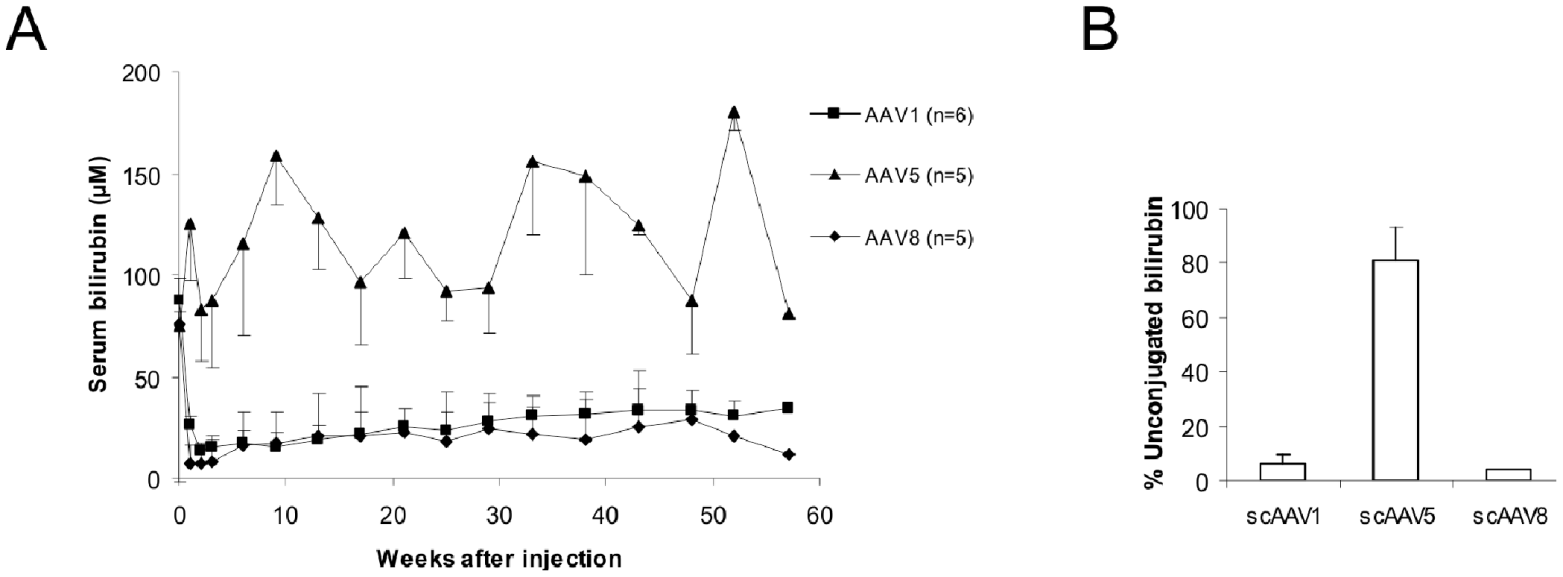
scAAV2/5 does not provide efficient correction of serum bilirubin in the Gunn rat. 3×10^11^ gc/kg scAAV-LP1-UGT1A1 serotype 1, 5 or 8 was administered via the portal vein. (A) Total serum bilirubin levels in AAV1 (▪); AAV5 (▴) and AAV8 (♦) treated rats. Data represent the mean and standard deviation per time point. The results obtained with AAV5 are statistically significant different (p<0.01) compared to the obtained with AAV1 and AAV8 using a mixed linear model analysis of variance. (B) Presence of unconjugated bilirubin in bile of treated rats as determined by HPLC. The results obtained with AAV5 are statistically significant different (p<0.001) compared to the obtained with AAV1 and AAV8 using the nonparametric Mann–Whitney test.

Serum billirubin levels were monitored for several months following an intraportal administration of 3×10^11^ gc/kg of scAAV-LP1-UGT1A1 vector pseudotyped with serotype 1, 5 and 8. Of these scAAV2/8 achieved the most prominent long term correction (Fig. 1). scAAV2/1 was also effective. In contrast, no correction was obtained with scAAV2/5. Even a portal vein injection of a 3 fold higher dose did not result in a significant reduction of serum bilirubin levels (Supplementary data). The significantly higher percentages of unconjugated bilirubin in bile compared to almost complete absence seen with the other serotypes, confirmed the lack of efficacy of this serotype (Fig. 1B).

### scAAV5 Vector Genomes Present in Gunn Rat Liver do not Express UGT1A1

**Figure 2:**
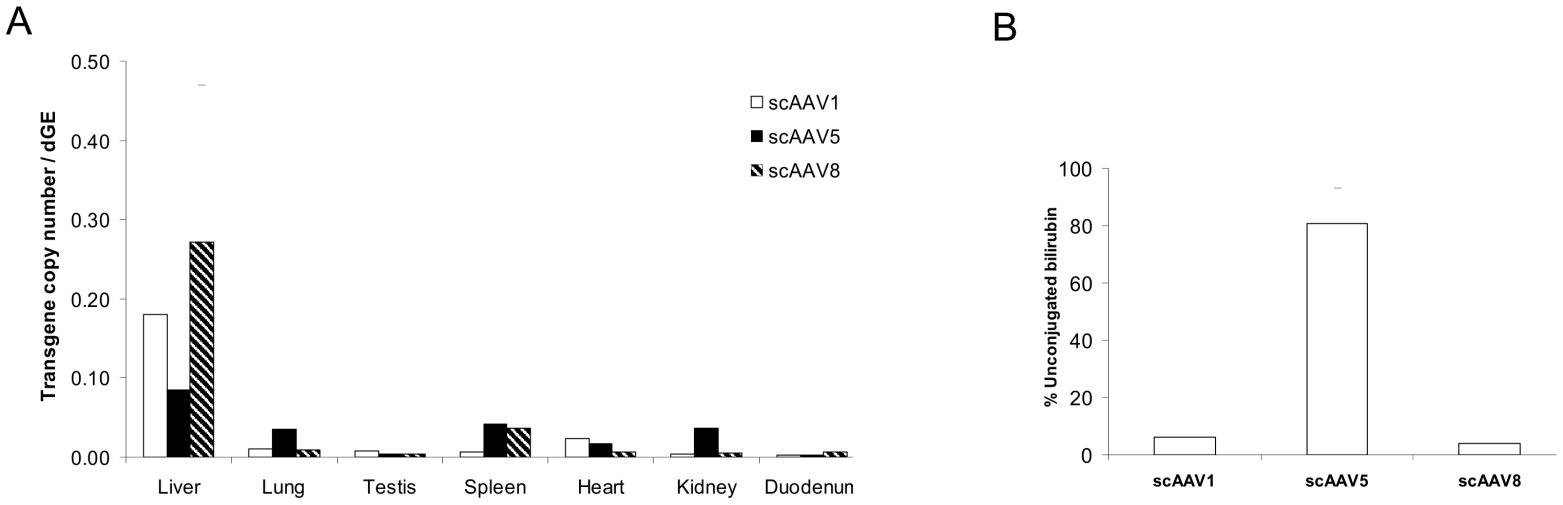
scAAV2/1, 5 and 8 vectors biodistribution and UGT1A1 mRNA expression levels in liver tissue. (A) UGT1A1 transgene copy number per diploid genome equivalent (dGE) in liver, lung, testis, spleen, heart, kidney and duodenum of male Gunn rats at 62 weeks after portal vein injection of scAAV1, 5 or 8 as calculated by the ratio of UGT1A1 copies and the rat β-actin gene copies in 100 ng of DNA as quantified by qPCR. (B) Relative UGT1A1 mRNA levels in liver quantified by qPCR and normalized with 18S rRNA levels. The results obtained with AAV5 are statistically significant different (p<0.001) compared to the obtained with AAV1 and AAV8 using the nonparametric Mann–Whitney test.

The presence of vector genomes was determined in liver, lung, kidney, heart, spleen, duodenum and testis (Fig. 2A). UGT1A1 genome copies per rat genome were normalized with the rat β-actin gene and based on a standard curve generated by dilution of UGT1A1 plasmid in rat genomic DNA. Tissue distribution of vector genomes revealed a strong liver tropism for scAAV8 and 1. In contrast, AAV5 displayed a broader tissue tropism its presence was detected in liver, spleen, kidney and lung (Fig. 2A). Besides cell receptor affinity, the prolonged periods in the circulation at higher levels, at least compared to AAV1, might contribute to its broader tropism (Figure S1).

The vector genomes delivered to the liver using AAV1 or AAV8 provided expression of UGT1A1 mRNA resulting in an efficient correction of serum bilirubin (Fig. 2B). In contrast, although the AAV5 vector did deliver genomes to the liver, their presence did not result in the expression of UGT1A1 mRNA explaining the lack of serum bilirubin correction. Increasing the vector dose 3-fold did not result in detectable UGT1A1 expression nor in correction of serum bilirubin (Figure S2).

### scAAV5 Transduces Liver in Rats Less Efficiently than in Mice

**Figure 3:**
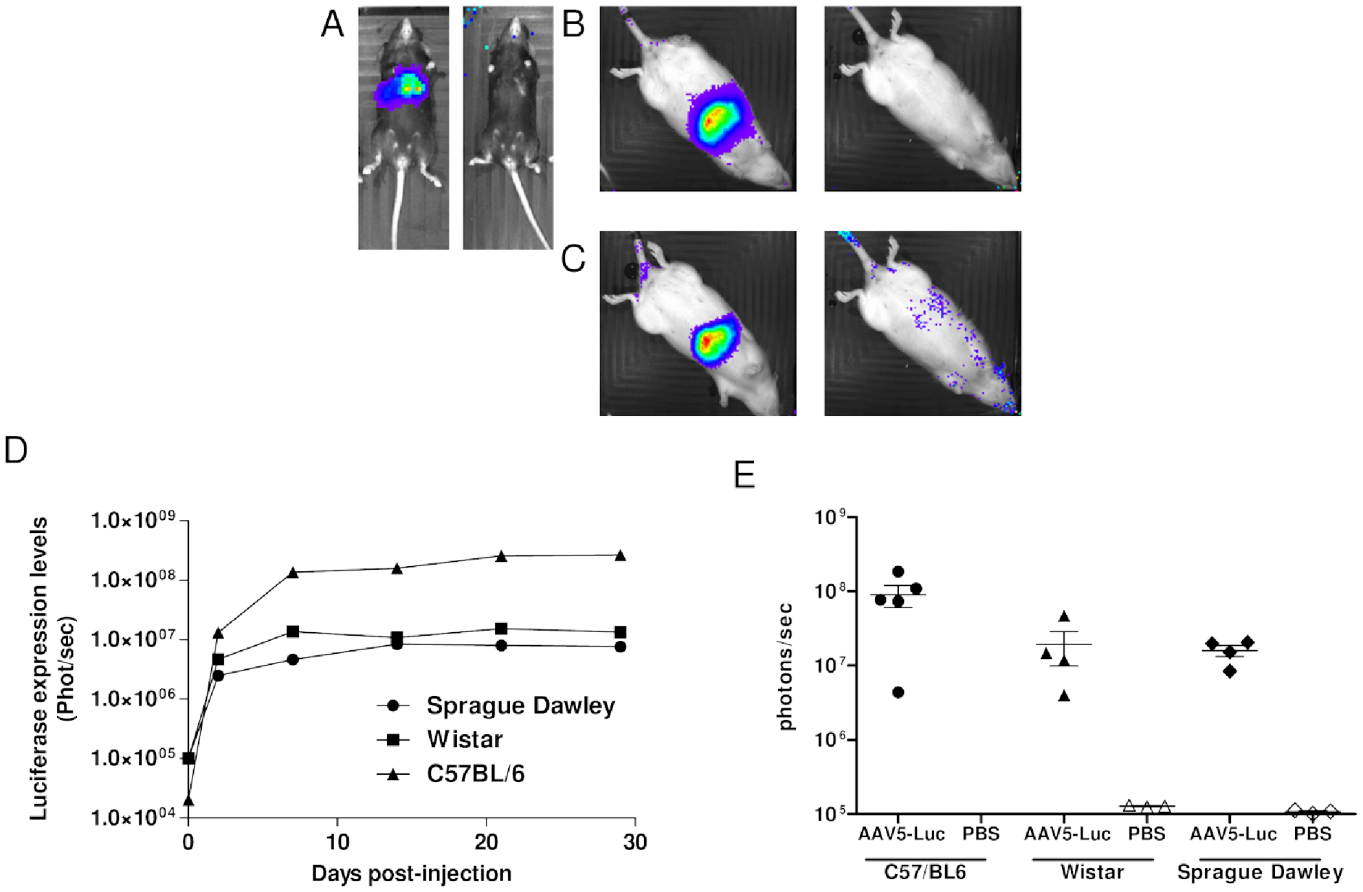
*In vivo* optical images of the CCD signal intensity in the liver of C57/BL6 female mice and Sprague Dawley/Wistar female rats injected with rAAV2/5-Ealb-hAAT-Luciferase-polyAPBGD-VD183 or PBS+/+ +5% sucrose controlled buffer matrix. (A) C57/BL6 mice, (B) Sprague Dawley rats and (C) Wistar rats all at day 30 after injection, (D) Luciferase expression levels measured in time (E) Luciferase expression at day 30 post-viral injection.

**Figure 4:**
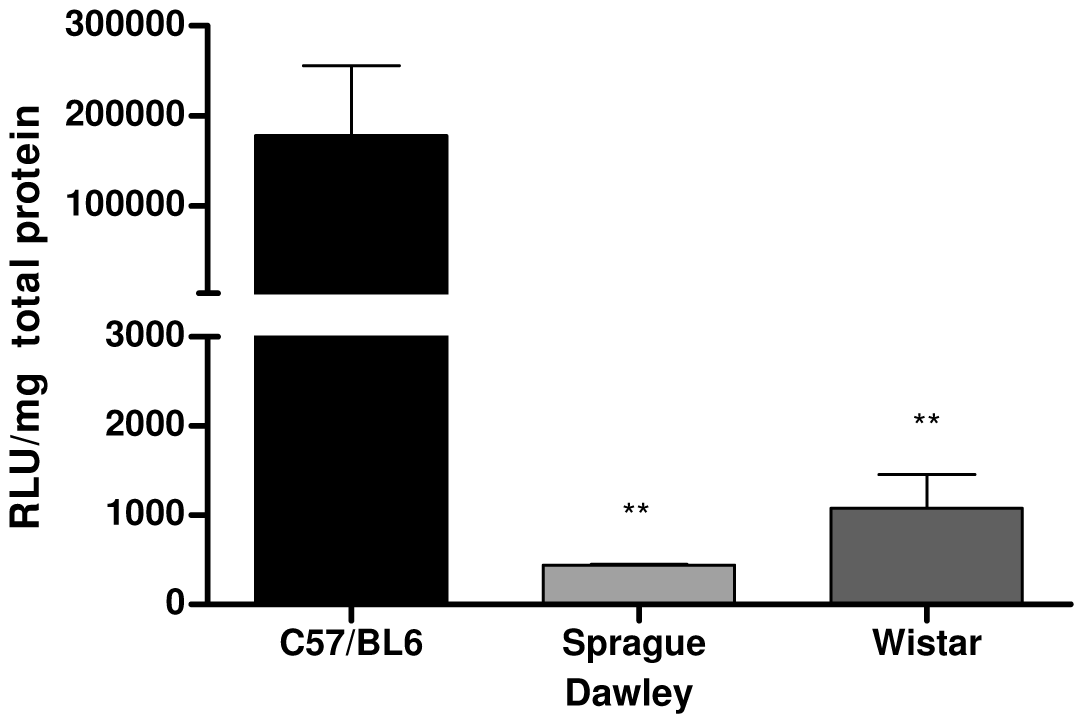
*Ex vivo* luciferase activity measured in the liver of C57/BL6 female mice and Sprague Dawley/Wistar female rats injected with rAAV2/5-Ealb-hAAT-Luciferase-polyAPBGD-VD183. All the animals were injected intravenously with 5×10^13^ GC/kg or an equivalent volume of PBS+/+ +5% sucrose controlled buffer matrix. Luciferase activity is represented as RLU per miligram of total protein. The results obtained in C57/BL6 mice are statistically significant different compared to the obtained in Sprague Dawley and Wistar rats using the nonparametric Mann–Whitney test (p<0.05 *, p<0.01 **, p<0.001 ***).

The lack of efficacy of AAV5 in the Gunn rat was unexpected. To exclude that this is due to some problem with the production of this serotype, the efficacy of a single batch was studied in a mouse model and two different rat strains. C57BL/6 mice, Sprague Dawley and Wistar rats were injected with a dose of 5×10^13^ gc/kg of an AAV5 vector ferrying a hepatocyte specific luciferase expression construct. The use of a hepatocyte specific promoter should confine the luciferase expression to the liver. As is shown in Fig. 3A to C, this was indeed the case in all three models. The emitted light in the mouse model appeared about five fold higher than in both rat strains confirming the lower efficacy of AAV 5 in rats (Fig. 3D,E). In all three models the luciferase expression remains stable after the initial rapid increase demonstrating that the lower efficacy in the rats is not caused by a loss of the transduced cells due to an adaptive immune response. Subsequent in vitro analysis showed that in the luciferase expression per mg of protein is about 100 fold higher in the mouse model compared to both rat models. This underscored the poor efficacy of AAV5 in rat liver (Fig. 4). Taking into account that the rat liver of is 10 times bigger than mouse liver the total luciferase activity determined *ex vivo* correlated with the luciferase activity measured *in vivo.* The smaller difference in emitted light was due to the 10 fold higher number of vectors injected in the rats to reach the same dose per kg. In addition the black fur and skin of the C57BL/6 mice may have shielded some of the light omitted by transduced hepatocytes.

## Discussion

AAV is the most promising delivery system for *in vivo* gene therapy as demonstrated by its efficacy in patients suffering from various inherited disorders [2]–[4]. Studies in non-human primates demonstrated that for liver transduction serotype 5 and 8 seem the best candidates [12]. Therefore the potential use of both for treating CN syndrome was studied in a relevant animal model, the Gunn rat. The efficacy of AAV5 and AAV8 was compared to that of AAV2/1, previously reported to have the highest efficiency in this model. Portal vein administration of AAV1 and 8 carrying a hepatocyte specific expression construct resulted in high expression of UGT1A1 and therapeutic correction of serum bilirubin levels. However, in contrast to these two the efficacy of AAV2/5 in this rat model was very low. Since in mice and non-human primates this serotype had been shown to be effective this poor efficacy in rats was unexpected. To exclude a problem with the AAV5 vector and/or the rat model used a direct comparison between two rat models and a mouse model was performed. This confirmed that the efficacy of AAV5 liver transduction is at least 100 fold lower in rats. The poor efficacy cannot be explained by lack of genome delivery to the liver since the vector genome levels in livers of AAV5 injected rats are comparable to those in AAV1 or AAV8 treated animals. However, the expression of UGT1A1 mRNA, in AAV5 injected rats is much lower. Since these vectors ferry identical hepatocyte specific expression constructs this poor expression seems to suggest that in rats AAV5 does not transduce hepatocytes but end up in a liver cell in which these promoter is inactive. To investigate this hypothesis, a dose of 3×10^11^ gc/kg of scAAV5-CMV-GFP (which is expressed in all cell types) was injected into the tail vein of eight Gunn rats. This also resulted in the presence of vector genomes mainly in liver and spleen, but not in detectable expression of GFP mRNA and protein, which renders this explanation unlikely (data not shown). In mice, the stronger liver tropism with AAV1 than AAV5 can be explained by its receptor, platelet derived growth factor receptor (PDGFR) which is expressed at high levels on hepatocytes [22]. However, their is not a single receptor for any of AAV serotypes, and the transduction of various cell types is dependent on the variable expression of these receptors on the cell membrane. For example, it has been reported that AAV5 transduction is highly dependent of alpha-2,3-sialic acid present in N-linked glycoproteins [23]. It might be that in rat hepatocytes the exposure of such molecules on the cell membrane is not optimal for AAV5 transduction. Also with the luciferase expression construct the transduction of rat liver was low compared to that in mice. Since with this vector also a hepatocyte specific promoter (Ealb) was used these data supported the observations in the Gunn rat. A possible explanation for the lack of expression could be an inefficient endocytosis of AAV5 since it makes use of two different entry pathways [24]. One of these entry pathways may for instance lead to the accumulation of AAV5 particles outside the nucleus such as the Golgi compartment [25].

The large difference in efficacy of AAV5 found between mice and rat is unexpected but recently two new species of AAV’s from rat and mouse liver [26] have been identified. These new AAV subtypes only infected there native animal species, thus the rat AAV only efficiently infected rat liver while the mouse AAV only infected mouse liver. This demonstrated that in rodents these respective AAVs have diverged substantially which may explain the large differences in efficacy observed. In this respect, the fact that of the human serotypes AAV5 is taxonomically the most similar serotype to these rodent AAVs seems of interest.

Recently it was reported that in rabbits AAV5 also transduced liver very poorly [27]. The mechanism for this lack of efficacy in this animal model and in rats is not elucidated and will require additional studies, for instance using AAV5 with a labeled capsid and/or genome. Knowledge about this mechanism would ultimately improve understanding of the cellular proteins and activities mediating rAAV5 transduction.

In conclusion, we showed that scAAV8 and scAAV1 provided an efficient liver transduction in the rat model for CN syndrome, the Gunn rat. Furthermore, our data indicated that proof of concept studies with AAV5 as a vector for correcting liver disorders are not possible in rat models.

## Supporting Information

Data S1 **Supplementary data.**
(PDF)
