# Correction: Adeno-Associated Viral Vector Serotype 5 Poorly Transduces Liver in Rat Models

**DOI:** 10.1371/journal.pone.0101857

**Published:** 2014-06-26

**Authors:** The PLOS ONE Staff

Figure 2 is incorrect. The authors have provided a corrected version here.

**Figure 2:**
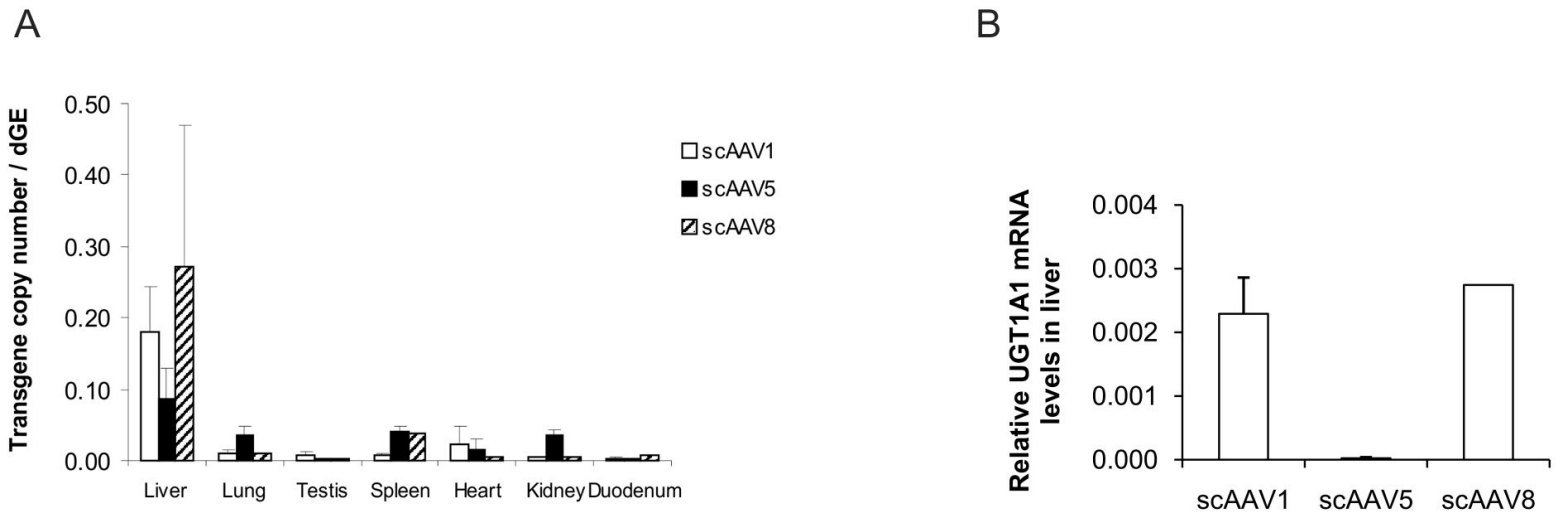
scAAV2/1, 5 and 8 vectors biodistribution and UGT1A1 mRNA expression levels in liver tissue. (A) UGT1A1 transgene copy number per diploid genome equivalent (dGE) in liver, lung, testis, spleen, heart, kidney and duodenum of male Gunn rats at 62 weeks after portal vein injection of scAAV1, 5 or 8 as calculated by the ratio of UGT1A1 copies and the rat β-actin gene copies in 100 ng of DNA as quantified by qPCR. (B) Relative UGT1A1 mRNA levels in liver quantified by qPCR and normalized with 18S rRNA levels. The results obtained with AAV5 are statistically significant different (p<0.001) compared to the obtained with AAV1 and AAV8 using the nonparametric Mann–Whitney test.
