# Correction: Adeno-Associated Viral Vector Serotype 5 Poorly Transduces Liver in Rat Models

**DOI:** 10.1371/journal.pone.0119777

**Published:** 2015-03-25

**Authors:** The PLOS ONE Staff

The sixth author’s name is spelled incorrectly. The correct name is Gloria Gonzalez-Aseguinolaza.

The correct citation is: Montenegro-Miranda PS, Pañeda A, ten Bloemendaal L, Duijst S, de Waart DR, Gonzalez-Aseguinolaza G, et al. (2013) Adeno-Associated Viral Vector Serotype 5 Poorly Transduces Liver in Rat Models. PLoS ONE 8(12): e82597. doi:10.1371/journal.pone.0082597
