# Supplementary material for: Adeno-Associated Viral Vector Serotype 5 Poorly Transduces Liver in Rat Models
Source: PLoS One. 2013 Dec 27;8(12):e82597. doi: 10.1371/journal.pone.0082597 (PMC3873922; doi:10.1371/journal.pone.0082597)
Supplement: Data S1 — Supplementary data. (PDF) [file pone.0082597.s001.pdf]

Supplementary data

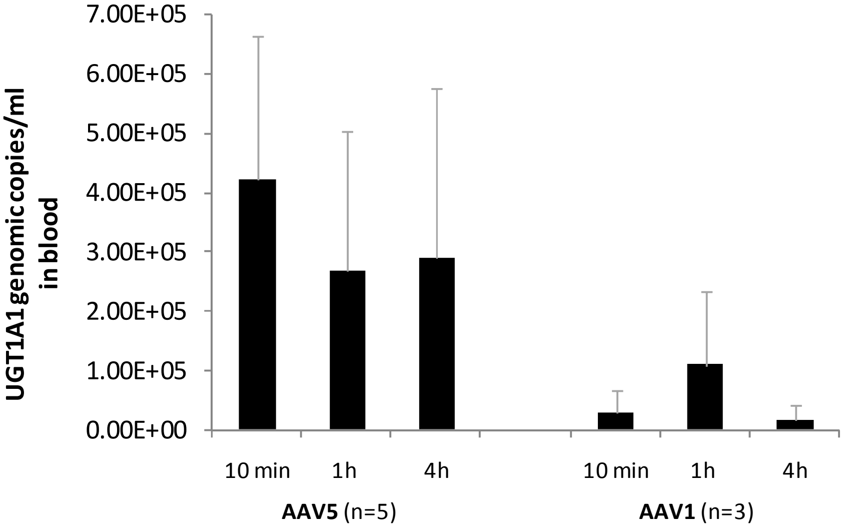

Figure S1 – Blood clearance upon intraportal vein injection of AAV5 and AAV1 in Gunn rats. Clearance of the vector from rat plasma was determined using a quantitative PCR (qPCR) assay on samples collected at different time points after portal vein administration of 3×10^{11} vg/kg scAAV2/5 or 1-LP1-UGT1A1. Results are expressed as mean transgene copy number (vg)/ml) ± SE.

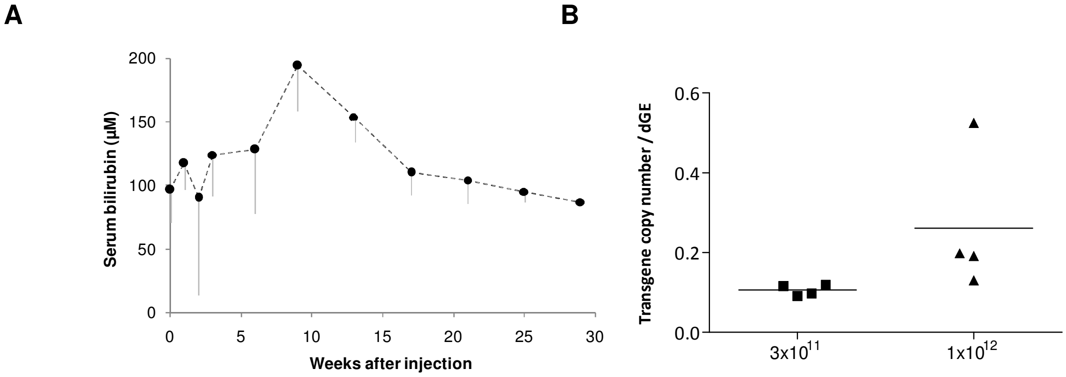

Figure S2 – Serum bilirubin levels and transgene copy numbers in liver of Gunn rat injected with scAAV2/5-LP1-UGT1A1. (A) Serum bilirubin levels of male Gunn rats were monitored for 28 weeks upon portal vein injection of $1x10^{12}$ gc/kg of scAAV2/5-LP1-UGT1A1. (B) UGT1A1 transgene copy number per diploid genome equivalent (dGE) in livers of treated animals, calculated using the ratio of UGT1A1 copies the rat β-actin gene copies in 100ng of DNA as quantified by qPCR.
